# Self-harm in young adolescents (12–16 years): onset and short-term continuation in a community sample

**DOI:** 10.1186/1471-244X-13-328

**Published:** 2013-12-02

**Authors:** Paul Stallard, Melissa Spears, Alan A Montgomery, Rhiannon Phillips, Kapil Sayal

**Affiliations:** 1 Department of Health, Child and Adolescent Mental Health Research Group, University of Bath, 22-23, Eastwood BA2 7AY, UK; 2 NIHR Research Methods Training Fellow, School of Social and Community Medicine, University of Bristol, Bristol, UK; 3 Medical Statistics and Clinical Trials, Nottingham Clinical Trials Unit, University of Nottingham, Nottingham, UK; 4 Institute of Primary Care & Public Health, Cardiff University, Cardiff, UK; 5 Division of Psychiatry and Applied Psychology, Institute of Menal Health, University of Nottingham, Nottingham, UK

**Keywords:** Self-harm, Young adolescents, Onset, Continuity

## Abstract

**Background:**

To investigate the prevalence of self-harm in young adolescents and factors associated with onset and continuity over a one year period.

**Method:**

Prospective longitudinal study. Participants were young adolescents (n = 3964) aged 12–16 years attending 8 secondary schools in the Midlands and South West of England.

**Results:**

Over a one year period 27% of young adolescents reported thoughts of self-harm and 15% reported at least one act of self-harm. Of those who self-harmed, less than one in five (18%) had sought help for psychological problems of anxiety or depression. Compared with boys, girls were at increased risk of developing thoughts (OR 1.61, 95% CI 1.26-2.06) and acts (OR 1.40, 95% CI 1.06-1.84) of self-harm, particularly amongst those girls in school year 9 (aged 13/14, thoughts adjusted Odds Ratio (aOR) 1.97, 95% CI 1.27-3.04; acts aOR 2.59, 95% CI 1.52-4.41). Of those reporting thoughts of self-harm at baseline, 60% also reported these thoughts at follow-up. Similarly 55% of those who reported an act of self-harm at baseline also reported that they had self-harmed at follow-up. Insecure peer relationships increased the likelihood that boys and girls would develop self-harming behaviours, as did being bullied for boys. Low mood was associated with the development of self-harming thoughts and behaviours for boys and girls, whilst a strong sense of school membership was associated with a reduced risk of developing thoughts of self-harm for boys and increased the likelihood of self-harming thoughts and behaviours ceasing for girls.

**Conclusion:**

Self harm in young adolescents is common with one in four reporting self-harming thoughts and one in six engaging in self-harming behaviour over a one year period. Self-harm is already established by 12/13 years of age and for over half of our sample, self-harming thoughts and behaviour persisted over the year. Secure peer and strong school relationships were associated with less self-harm. Few seek help for psychological problems, suggesting a need to increase awareness amongst all professionals who work with young adolescents about self-harm and associated risk factors.

## Background

Estimated prevalence rates of self-harm in adolescence vary and are influenced by the definition applied, cohort assessed and method of measurement [1]. Despite these methodological differences, it is clear that self-harm in adolescents is a significant problem with community surveys indicating that between 3-10% report at least one episode of self-harm in the past year, with lifetime rates of between 9-14% [1-6]. Approximately half of adolescents who self-harm will do so more than once; cutting and overdose are the main methods used [3,6] and comparatively few self-harming episodes will result in presentation at hospital [3,5,7]. A further 15-22% of young people report thoughts of self-harm over the past year which they did not act upon [1-3,5,7]. However, there is a strong association between self-harming thoughts and non-suicidal self-injury and suicide attempts in both community [1] and clinical groups [8,9].

Cross-sectional studies have identified a number of factors associated with self-harm in adolescents. Self-harm has been found to be correlated with female gender [1,3], low self-esteem in girls [10], illegal drug use [1,10,11] smoking [1], being bullied [10], not living with both parents [1] low social economic status [1] and knowing a friend who had self-harmed [10]. Longitudinal studies have identified risk factors associated with the development of self harm which include mental health problems of anxiety [4,12-14], depression [4,12,14,15], high risk alcohol use [4], cannabis use [4] and substance misuse [14]. Less is known about protective factors, although parents living together and being supportive appear helpful [16-18].

Several prospective longitudinal community studies have assessed self-harm in adolescence from age 14 years through to young adulthood [4,14,19]. However little is known about self-harm in younger adolescents (≤14 years) and factors associated with onset or short-term continuation or cessation during this particularly vulnerable time. In an exploratory study, Hankin & Abela assessed self-harm in 103 young adolescents aged 11–14 [20]. 18% of young adolescents self-harmed over the 2½ year follow-up with 14% being new cases. Self-harm was associated with negative cognitive style, depressive symptoms and lack of social support. Whilst half of those who self-harmed at baseline continued to self-harm at follow-up it was not possible to investigate factors associated with cessation or continuation. In a Norwegian study, Larsson & Sund assessed 2,464 adolescents aged 12–15 years over a one year period [21]. Lifetime rates of self-harm without suicidal intent were 3%. Those who self-harmed were more likely to be girls, have more depressive symptoms and somatic problems, smoke cigarettes and know someone who had self-harmed. In Sweden, Lundh et al. assessed a community sample of 1052 young adolescents aged 13–15 years twice over a two year period [22]. Using a 9-item self-harm inventory 45.1% of girls and 37.9% of boys reported at least one episode of self-harm during the past 6 months. Depressive symptoms at time one predicted the incidence of new cases of repeated self-harm at time two. Subsequent analyses explored risk factors for developing self-harm [23,24]. There was evidence of a bi-directional relationship between depressive symptoms and repeated self-harm in girls but not boys [23]. Poor sleep was also a risk factor for the development of self-harm over a one year period [24]. In terms of remission, 22.7% of those who engaged in repeat self-harm at baseline were no longer self-harming one year later [23].

The above indicates that self-harm is evident in younger adolescents although research with this younger group is still limited. To address gaps in knowledge this paper reports on the prevalence of self-harming thoughts and behaviours in a community sample of young adolescents aged 12–16 in the UK and factors associated with their onset and continuation over a twelve month period.

## Method

### Participants

Participants were young adolescents in school years 8–11 (aged 12–16 years) attending 8 mixed sex non-denominational schools in the Midlands and South West of England. These schools were participating in a randomised controlled trial (PROMISE) evaluating the effectiveness of school based interventions on symptoms of depression [25]. Adolescents in these year groups were eligible to participate unless they were not attending school (for example, due to long term sickness, being excluded from school, or educated elsewhere) or did not participate in Personal Social and Health Education (PSHE) lessons in which the assesments were conducted.

Participation in the trial required consent from the school head teacher and the child’s parent and signed assent from the adolescent. The study was approved by the University of Bath School for Health Ethics Committee.

### Measures

Information was collected from self-report questionnaires completed during class at assessment 1 (baseline) and assessment 2 (6 months later). The primary outcomes investigated in this paper are the frequency of self-harming thoughts and behaviours. Other variables found to be associated with the development or continuation of self-harm, or are known confounding variables which need to be adjusted for, were extracted from the data set and analysed. These included socio demographic variables (i.e. gender, family structure, age, socio-economic status), mood, behavioural risk factors (alcohol, cannabis and other drug use, bullying), relationships and support (i.e. peers and school). The specific items are detailed below.

#### Self-harming thoughts and behaviours

Self-harm was assessed by two questions taken from the Avon Longitudinal Study of Parents and Children (ALSPAC) study (Life of a teenager 16+ assessment; (see http://www.alspac.bris.ac.uk). The first was a slightly modified question that assessed thoughts: “have you ever thought about hurting yourself, even if you would not really do it, in the last 6 months?” (categorised as not at all vs. once or more). The second assessed self-harming behaviour: “have you ever hurt yourself on purpose in any way (e.g. by taking an overdose of pills or by cutting yourself), in the last 6 months?” (categorised as not at all vs. once or more).

#### Socio-demographic variables

School year group, gender, ethnicity (white vs. other), who the participant lives with (both parents, parent and new partner, single parent, other), and the four items that comprise the Family Affluence Scale [26], i.e. family own a car; young adolescent has their own room; been on holiday in the last year; family own a computer, were assessed.

#### Depressed mood

Mood was assessed by the Short Mood and Feelings Questionnaire (SMFQ) [27]. The 13-item SMFQ has been used with community and clinical samples. It correlates well with other measures of depression and has good test/re-test reliability. On the basis of total scores participants were either classified as having low (<5) or high symptoms (≥5) [25].

#### Behavioural risk factors

Participants were asked about their drinking of alcohol (categorised as never or once or twice in the past 6 months vs. more than 2–4 times a month), use of cannabis (categorised as never vs. used at least once), and use of other street drugs (categorised as never vs. used at least once) over the past 6 months. They were asked whether they had been bullied (categorised as never, once or twice vs. more than 2–3 times per month) and if they had taken part in bullying others (categorised as never vs. once or twice or more than 2–3 times per month) over the preceding 6 months.

#### Peer attachment

The Attachment Questionnaire for Children [28,29] asked participants to select one of three statements to describe their relationship with their peers (secure, avoidant or ambivalent attachment style).

#### School connectedness

The degree to which participants felt accepted, valued, respected and included in their school was assessed by a short 8-item version of the Psychological Sense of School Membership scale [30]. Items were summed with total scores of (≤24) being classified as low and (>24) as high.

#### Help-seeking

In addition to the above risk factors participants were asked whether they had seen their GP or another professional over the previous 6 months for help with anxiety or depression.

### Statistical analysis

Four separate analyses were undertaken using Stata version 11.

1) Prevalence at baseline and incidence at follow up of self-harming thoughts and behaviours were calculated.

2) For those who did not report any self-harm at the first assessment, factors associated with the development of self-harm at follow-up were investigated.

3) For those who did report self-harm at the first assessment, factors associated with continuation of self-harm at follow-up were investigated.

4) The association between self-harming thoughts and behaviours was investigated.

For analyses (1–3) the frequency of absence/presence of self-harm at assessment 1 (baseline) and at assessment 2 (6 months follow-up) were calculated. The corresponding adjusted odds ratios were then calculated separately for males and females.

For analysis (4), numbers of participants reporting neither self-harm thoughts nor behaviour at baseline but going on to report one or both at 6 months are described. An odds radio was calculated to describe the association between reporting only self-harming thoughts at baseline and going on to report self-harm behaviour at 6 months, compared to reporting neither thoughts nor behaviours at baseline.

All odds ratios were adjusted initially for baseline age and trial arm. We adjusted for trial arm in our analyses as the data are from a randomised controlled trial; however, there was no evidence that the trial intervention had any effect on either depression or self-harm behaviour at follow-up ^32^. We then adjusted for all other variables in multivariable logistic regression models. Multi-level models with year group as a level (being the trial unit of randomisation) were considered to take account of clustering within the data. This made no material difference to the estimates and their standard errors so the data presented are those from simpler logistic regression models. The Intra-Class Correlations (ICCs) were as follows: school ICC = 0.005; year group ICC = 0.0012; class ICC = 0.08.

Comparisons of baseline data were carried out for those with vs. without missing self-harm data at either time point. Sensitivity analyses were conducted to assess the potential effect of these missing data. At most, 14% of individuals had missing information on self-harm thoughts or behaviour at either assessment. Because missing values were not likely to be missing at random, and to avoid any loss in efficiency, missing values for self-harm status were imputed using multiple imputations by chained equations. Twenty imputed datasets were created using imputations based on variables predictive of missing status including gender, age, alcohol, cannabis & drugs use, bullying others and measures of depression, school connectedness & peer attachment. The ICE set of commands within STATA 11 were used. For the majority of associations between self-harm and risk factors investigated the imputations made no material difference to our overall conclusions and so the estimates from simple logisitic regression models are presented within the paper. The imputations did in some cases strengthen the possibility of an association with self-harm status, however in all cases this was a very small change in the lower limit of the confidence interval and was only in the case of the simple adjusted odds ratios Simple and adjusted odds ratios are reported.

## Results

### Sample

A summary of school demographics of study participants compared with national figures is presented in Table 1.

**Table 1:** Comparison between study schools and national data

**School**	**Pupils in trial**	**% White**^**1**^	**% Special needs without statements**^**1**^	**Overall absence**^**2**^	**Persistent absence**^**2**^	% achieving 5 A*-C passes including level 2 English and Math’s^**3**^	**% Eligible for free school meals**^**1**^
1	710	63.8%	20.2%	5.4%	2.7%	69%	11.9%
2	623	89.7%	8.1%	8.3%	7.7%	61%	7.0%
3	835	93.2%	17.9%	6,5%	5.1%	55%	9.5%
4	783	95.3%	6.9%	6.1%	2.8%	75%	2.5%
5	848	77.6%	36.0%	11.1%	13.8%	36%	31.2%
6	530	83.5%	10.5%	4.7%	1.7%	No year 11	3.3%
7	534	92.8%	10.1%	7.7%	6.2%	47%	7.6%
8	167	98.7%	19.7%	7.9%	7.4%	48%	9.2%
Total trial	5030	85,5%	16.8%	7.3%	6.0%	56.7%	11.2%
National average		81.2%	19.7%	6.s9%	4.4%	50%	15.4%

^1^National Stats: Statistical first release: Schools. Pupils and their characteristics: January 2010: Department for Education, 13/05/2010.

^2^National Stats: Statistical first release: Pupil absence in schools in England including pupil characteristics: 2009/10. Department for Education, 29/03/11.

^3^Achievement and attainment tables 2009; Department for Education.

The average of our 8 schools in terms of ethnicity, deprivation (eligibility for school free meals), pupil absence rates and academic ability (examination results and proportion of children with identified special educational needs) was similar to national rates although the range of our sample is narrow. For example only one school had higher free school meal eligibility and lower rates of academic attainment (Table 2).

**Table 2:** Self-harming thoughts and behaviours by gender and year group

	**Boys**					**Girls**					**Overall**
	**Yr 8**	**Yr 9**	**Yr 10**	**Yr 11**	**Overall**	**Yr 8**	**Yr 9**	**Yr 10**	**Yr11**	**Overall**	
						**First assessment**					
**Thoughts about hurting yourself in past 6 months**	117/652 (18%)	77/628 (12%)	89/598 (15%)	54/405 (13%)	337/2283 (15%)	138/628 (22%)	135/639 (21%)	167/590 (28%)	103/376 (27%)	543/2233 (24%)	880/4516 (19%)
**Have you ever hurt yourself on purpose in past 6 months**	47/651 (7%)	34/625 (5%)	53/598 (9%)	382/406 (6%)	158/2280 (7%)	61/630 (10%)	55/636 (9%)	105/591 (18%)	56/378 (15%)	277/2235 (12%)	435/4515 (10%)
						**Second assessment**					
**Have you ever thought about hurting yourself in past 6 months**	108/652 (17%)	76/611 (12%)	91/561 (16%)	41/362 (11%)	316/2186 (14%)	121/620 (20%)	161/632 (25%)	163/567 (29%)	76/334 (23%)	521/2153 (24%)	837/4339 (19%)
**Have you ever hurt yourself on purpose in past 6 months**	58/653 (9%)	43/609 (7%)	47/560 (8%)	24/359 (7%)	172/2181 (8%)	60/617 (10%)	93/629 (15%)	110/567 (19%)	38/331 (11%)	301/2144 (14%)	473/4325 (11%)
						**Incident self-harm**					
**New thoughts at second assessment**	36/492 (7%)	35/482 (7%)	31/436 (7%)	20/279 (7%)	122/1689 (7%)	37/449 (8%)	69/465 (15%)	45/375 (12%)	17/216 (8%)	168/1505 (11%)	290/3194 (9%)
**New behaviour at second assessment**	30/555 (5%)	26/518 (5%)	24/468 (5%)	14/304 (5%)	94/1845 (5%)	22/516 (4%)	53/534 (10%)	31/431 (7%)	15/253 (6%)	121/1734 (7%)	215/3579 (6%)

A total of 5030 (91.5% of the eligible sample) consented to take part in the trial and of these, 3964 (78.8%) completed questions relating to thoughts of self-harm at both assessments. Questions assessing self-harming behaviours were completed by 3955 (78.6%) at both time points.

A comparison of those who did and did not provide data about self-harm (thoughts and behaviour) at baseline and 6 months with regards to relevant baseline variables, is summarised in Additional file 1.

Non-availability of self-harm data was associated with: older year group (Year 11); who the participant lived with; high SMFQ score; alcohol and cannabis use; bullying others; family not owning a car; and low school membership. These variables were all included within the multiple imputation process.

### Prevalence of self-harming thoughts and behaviours

Similar proportions of young adolescents reported self-harming thoughts over the preceding six months at assessment 1 (19.5%) and assessment 2 (19.3%). At least one act of self-harm over a six month period was reported by 9.6%-10.9% of participants. Cumulative rates indicated that 27% (1060/3964) of young adolescents experienced thoughts of self-harm and 15% (591/3955) reported acts of self-harm at some stage over the 12 month period. The overall prevalence of help-seeking in the past 6 months for problems of anxiety or depression was 6%.

Overall rates of self-harming thoughts across both time points by year group were: year 8 = 26%, year 9 = 25%, year 10 = 28% and year = 24%. For self-harming behaviour rates were; year 8 = 13%, year 9 = 13%, year 10 = 18%, year 11 = 14%.

### The development of self-harm

A total of 3194 (80.5%) young adolescents reported no thoughts of self-harm in the 6 months preceding the first assessment. When they were assessed at time 2, the incidence of self-harming thoughts was 9.1% (n = 290) with more girls (11.2%) than boys (7.2%) (OR = 1.61, 95% CI 1.26-2.06) reporting thoughts of self-harm.

Of those who did not report any self-harming behaviours (n = 3579) at the first assessment, 6.0% (n = 215) had self-harmed by the second assessment with girls reporting more acts of self-harm than boys (OR = 1.40, 95% CI 1.06-1.84).

#### Socio-demographic factors

The role of age, ethnicity, family living arrangements and family affluence in the development of self-harm was investigated. After adjusting for other socio-demographic variables, only age and not going away on holiday (an indicator of affluence) were associated with self-harm. Girls in school year 9 (aged 13/14 years) had increased odds of developing self-harming thoughts (aOR = 1.97, 95% CI 1.27-3.04) and behaviours (aOR = 2.59, 95% CI 1.52-4.41) by the second assessment, compared to girls in school year 8. Boys (aOR = 1.86, 95% CI 1.10-3.13) and girls (aOR = 2.05, 95% CI 1.28-3.27) who had not been on holiday in the past year had increased odds of developing self-harming behaviours. This also increased the odds of developing self-harming thoughts for girls (aOR = 1.94, 95% CI 1.27-3.03).

#### Mood and behavioural risk factors

The relationship between baseline mood, alcohol, cannabis, and drug use, bullying, peer attachment, school connectedness and the onset of self-harm at assessment 2 is summarised in Table 3.

**Table 3:** Mood, substance use, bullying and school connectedness in the development of self-harm for boys and girls

**Boys**							
**Variable**	**Level**	**No self-harm thoughts at time 1**	**Developed self-harm thoughts at time 2**	Adjusted OR* (95% CI)	**Overall p-value**	Adjusted OR* (95% CI) from MI	**Multiple adjusted OR**^T^**(95% CI)**
**Alcohol**	**Never/1 or 2x**	1399	88	1.0	<0.001	1.0	1.0
	**2/4 + per mth**	277	32	2.26 (1.43, 3.57)		2.09 (1.34, 3.28)	1.56 (0.91, 2.67)
**Cannabis**	**Never used**	1556	103	1.0	<0.001	1.0	1.0
	**Used**	119	18	2.84 (1.61, 4.99)		2.59 (1.52, 4.41)	2.24 (1.12, 4.48)
**Drugs**	**Never used**	1660	119	1.0	0.467	1.0	1.0
	**Used**	18	2	1.74 (0.39, 7.73)		2.14 (0.55, 8.36)	0.57 (0.11, 2.92)
**Bullied**	**Never/1 or 2x**	1609	107	1.0	<0.001	1.0	1.0
	**2/3 + per mth**	72	14	3.40 (1.83, 6.32)		3.11 (1.70, 5.67)	1.82 (0.84, 3.94)
**Bullying others**	**Never**	1379	88	1.0	0.002	1.0	1.0
	**Regularly**	290	34	1.94 (1.27, 2.96)		1.81 (1.17, 2.80)	1.29 (0.79, 2.10)
**Peer attachment**	**Secure**	1392	88	1.0	<0.001	1.0	1.0
	**Avoidant**	75	10	2.25 (1.12, 4.55)		2.11 (1.06, 4.20)	1.43 (0.67, 3.06)
	**Anxious**	59	12	3.77 (1.93, 7.38)		3.02 (1.57, 5.81)	1.75 (0.79, 3.89)
**Baseline MFQ**	**0 – 4**	1481	83	1.0	<0.001	1.0	1.0
	**5+**	202	38	4.01 (2.63, 6.10)		4.08 (2.78, 5.98)	2.82 (1.71, 4.65)
**Baseline School connectedness**	**≤24**	182	26	1.0	<0.001	1.0	1.0
	**25+**	1502	96	0.41 (0.26, 0.66)		0.41 (0.26, 0.64)	0.55 (0.31, 0.95)
**Alcohol**	**Never/1 or 2x**	1536	73	1.0	0.053	1.0	1.0
	**2/4 + per mth**	295	21	1.68 (0.99, 2.86)		1.60 (0.97, 2.63)	1.29 (0.69, 2.41)
**Cannabis**	**Never used**	1698	81	1.0	0.022	1.0	1.0
	**Used**	132	12	2.16 (1.12, 4.17)		2.17 (1.12, 4.19)	1.21 (0.50, 2.89)
**Drugs**	**Never used**	1806	87	1.0	<0.001	1.0	1.0
	**Used**	23	6	7.47 (2.84, 19.66)		5.37 (1.97, 14.63)	5.95 (1.75, 20.25)
**Bullied**	**Never/1 or 2x**	1726	76	1.0	<0.001	1.0	1.0
	**2/3 + per mth**	109	18	4.28 (2.45, 7.50)		3.66 (2.09, 6.42)	2.42 (1.21, 4.86)
**Bullying others**	**Never**	1482	60	1.0	<0.001	1.0	1.0
	**Regularly**	337	31	2.37 (1.51, 3.74)		2.17 (1.39, 3.38)	1.68 (0.99, 2.85)
**Peer attachment**	**Secure**	1491	60	1.0	<0.001	1.0	1.0
	**Avoidant**	104	14	3.70 (1.99, 6.89)		2.88 (1.59, 5.20)	2.19 (1.07, 4.49)
	**Anxious**	84	9	2.87 (1.37, 6.01)		2.76 (1.32, 5.78)	1.16 (0.47, 2.86)
**Baseline MFQ**	**0 – 4**	1533	53	1.0	<0.001	1.0	1.0
	**5+**	305	41	4.38 (2.85, 6.73)		3.98 (2.71, 5.85)	2.24 (1.29, 3.87)
**Baseline School connectedness**	**≤24**	238	28	1.0	<0.001	1.0	1.0
	**25+**	1603	66	0.33 (0.20, 0.52)		0.32 (0.19, 0.54)	0.58 (0.32, 1.06)
**Girls**							
**Variable**	**Level**	**No self-harm thoughts at time 1**	**Developed self-harm thoughts at time 2**	Adjusted OR* (95% CI)	**Overall p-value**	Adjusted OR* (95% CI) from MI	**Multiple adjusted OR**^T^**(95% CI)**
**Alcohol**	**Never/1 or 2x**	1271	134	1.0	0.064	1.0	1.0
	**2/4 + per mth**	226	33	1.50 (0.98, 2.32)		1.61 (1.06, 2.45)	1.32 (0.80, 2.17)
**Cannabis**	**Never used**	1416	154	1.0	0.158	1.0	1.0
	**Used**	82	13	1.58 (0.84, 3.00)		1.63 (0.86, 3.09)	1.59 (0.74, 3.40)
**Drugs**	**Never used**	1485	167	1.0	0.541	1.0	1.0
	**Used**	16	1	0.53 (0.07, 4.08)		0.87 (0.15, 5.08)	0.23 (0.03, 2.03)
**Bullied**	**Never/1 or 2x**	1431	154	1.0	0.067	1.0	1.0
	**2/3 + per mth**	67	12	1.83 (0.96, 3.51)		2.04 (1.14, 3.64)	0.98 (0.43, 2.24)
**Bullying others**	**Never**	1350	149	1.0	0.700	1.0	1.0
	**Regularly**	148	18	1.11 (0.66, 1.87)		1.27 (0.78, 2.07)	0.59 (0.31, 1.10)
**Peer attachment**	**Secure**	1255	130	1.0	0.037	1.0	1.0
	**Avoidant**	100	17	1.76 (1.01, 3.07)		1.87 (1.08, 3.22)	1.07 (0.57, 1.99)
	**Anxious**	63	11	1.84 (0.94, 3.62)		2.44 (1.26, 4.73)	1.13 (0.55, 2.31)
**Baseline MFQ**	**0 – 4**	1127	87	1.0	<0.001	1.0	1.0
	**5+**	377	81	3.36 (2.41, 4.70)		3.67 (2.68, 5.04)	3.30 (2.25, 4.84)
**Baseline School connectedness**	**≤24**	195	37	1.0	<0.001	1.0	1.0
	**25+**	1294	128	0.47 (0.31, 0.70)		0.45 (0.31, 0.66)	0.75 (0.46, 1.21)
**Alcohol**	**Never/1 or 2x**	1463	93	1.0	0.038	1.0	1.0
	**2/4 + per mth**	263	27	1.65 (1.03, 2.66)		1.63 (1.06, 2.52)	1.03 (0.59, 1.82)
**Cannabis**	**Never used**	1637	104	1.0	<0.001	1.0	1.0
	**Used**	91	17	3.33 (1.84, 6.03)		2.76 (1.61, 4.73)	2.92 (1.37, 6.25)
**Drugs**	**Never used**	1711	118	1.0	0.263	1.0	1.0
	**Used**	20	3	2.06 (0.58, 7.29)		2.65 (0.89, 7.90)	0.53 (0.10, 2.69)
**Bullied**	**Never/1 or 2x**	1615	104	1.0	0.002	1.0	1.0
	**2/3 + per mth**	112	16	2.49 (1.41, 4.40)		2.51 (1.50, 4.18)	0.98 (0.49, 1.96)
**Bullying Others**	**Never**	1534	96	1.0	0.002	1.0	1.0
	**Regularly**	192	24	2.13 (1.32, 3.43)		2.16 (1.36, 3.45)	1.19 (0.68, 2.09)
**Peer attachment**	**Secure**	1389	75	1.0	<0.001	1.0	1.0
	**Avoidant**	144	17	2.30 (1.32, 4.02)		2.37 (1.38, 4.08)	1.15 (0.61, 2.16)
	**Anxious**	97	20	4.58 (2.65, 7.90)		4.67 (2.71, 8.01)	2.72 (1.49, 4.99)
**Baseline MFQ**	**0 – 4**	1201	42	1.0	<0.001	1.0	1.0
	**5+**	532	79	4.84 (3.26, 7.17)		5.33 (3.65, 7.78)	3.63 (2.28, 5.78)
**Baseline School connectedness**	**≤24**	266	40	1.0	<0.001	1.0	1.0
	**25+**	1453	78	0.32 (0.21, 0.48)		0.34 (0.23, 0.50)	0.62 (0.38, 1.02)

*ORs adjusted for age and trial arm.

^T^ORs adjusted for age, trial arm and all other variables.

MI = Multiple Imputation.

Symptoms of low mood increased the risk of developing self-harming thoughts and behaviours. Boys and girls who scored ≥5 on the SMFQ at assessment 1 were 2–3 times more likely to develop self-harming thoughts or behaviours over the next 6 months than those with fewer symptoms (<5).

After adjustment for all other mood and behavioural risk factors, alcohol use was not related to the development of self-harming thoughts or behaviours whereas cannabis use increased the odds that boys would develop self-harming thoughts and that girls would engage in acts of self-harm. Finally, although numbers are small and confidence intervals wide, boys who used street drugs were almost 6 times more likely to develop self-harming behaviour.

Boys who reported avoidant peer attachments and girls who reported anxious peer attachments were twice as likely to develop self-harming behaviours. Boys who were bullied were at a similar risk of engaging in self-harming acts whilst a strong sense of school membership had a protective function and reduced the odds of boys developing thoughts of self-harm.

### Continuation of self-harming

At the first assessment, 770 (19.4%) participants reported that they had experienced thoughts of self-harm at least once over the preceding 6 months. When assessed 6 months later, 458 (59.5%) continued to report self-harming thoughts, with girls being less likely than boys to report that they had stopped (OR = 1.42, 95% CI 1.06-1.91).

Of the 376 (9.5%) participants reporting self-harm behaviour at the first assessment, 207 (55.1%) continued to report self-harming acts when assessed six months later. This was more likely for boys than girls (OR = 2.11, 95% CI 1.38-3.26).

#### Socio-demographic factors

After imputing missing data there was no evidence that any socio-demographic variables were strongly associated with the cessation of self-harming thoughts/behaviours for boys or girls.

#### Mood and behavioural risk factors

The continuation of self-harming thoughts/behaviours and the relationship with mood, alcohol, cannabis and drug use, bullying and school connectedness is summarised in Table 4.

**Table 4:** Substance use, bullying and school connectedness and the continuation of self-harming thoughts/behaviour, for boys and girls

**Boys**							
**Variable**	**Level**	**Self-harm thoughts at time 1**	**No self-harm thoughts at time 2**	Adjusted OR* (95% CI)	**Overall p-value**	Adjusted OR* (95% CI) from MI	**Multiple adjusted OR**^T^**(95% CI)**
**Alcohol**	**Never/1 or 2x**	193	107	1.0	0.515	1.0	1.0
	**2/4 + per mth**	103	52	0.85 (0.51, 1.40)		1.07 (0.62, 1.83)	0.53 (0.29, 0.98)
**Cannabis**	**Never used**	228	117	1.0	0.102	1.0	1.0
	**Used**	69	42	1.63 (0.91, 2.91)		1.89 (1.09, 3.28)	1.99 (0.98, 4.05)
**Drugs**	**Never used**	282	151	1.0	0.285	1.0	1.0
	**Used**	17	11	1.77 (0.62, 5.05)		2.12 (0.76, 5.86)	1.50 (0.47, 4.76)
**Bullied**	**Never/1 or 2x**	240	120	1.0	0.005	1.0	1.0
	**2/3 + per mth**	61	43	2.38 (1.30, 4.37)		2.44 (1.35, 4.42)	2.12 (1.09, 4.15)
**Bullying others**	**Never**	203	101	1.0	0.021	1.0	1.0
	**Regularly**	97	62	1.80 (1.09, 2.97)		1.72 (1.06, 2.80)	1.57 (0.92, 2.67)
**School connectedness**	**≤24**	136	86	1.0	0004	1.0	1.0
	**25+**	161	75	0.50 (0.31, 0.80)		0.44 (0.28, 0.69)	0.66 (0.40, 1.09)
**Alcohol**	**Never/1 or 2x**	74	33	1.0	0.698	1.0	1.0
	**2/4 + per mth**	64	28	1.15 (0.56, 2.39)		1.37 (0.68, 2.73)	0.93 (0.40, 2.14)
**Cannabis**	**Never used**	95	38	1.0	0.060	1.0	1.0
	**Used**	42	22	2.19 (0.97,4.95)		2.32 (1.05, 5.16)	1.90 (0.72, 4.98)
**Drugs**	**Never used**	128	56	1.0	0.242	1.0	1.0
	**Used**	11	6	2.20 (0.59, 8.30)		2.66 (0.79, 9.02)	1.73 (0.39, 7.58)
**Bullied**	**Never/1 or 2x**	110	45	1.0	0.155	1.0	1.0
	**2/3 + per mth**	30	17	1.82 (0.80, 4.13)		2.10 (0.92, 4.84)	1.95 (0.77, 4.94)
**Bullying others**	**Never**	89	37	1.0	0.262	1.0	1.0
	**Regularly**	49	25	1.50 (0.74, 3.06)		1.32 (0.66, 2.64)	1.15 (0.52, 2.54)
**School connectedness**	**≤24**	67	34	1.0	0.154	1.0	1.0
	**25+**	73	28	0.61 (0.31, 1.20)		0.46 (0.24, 0.87)	1.07 (0.48, 2.39)
**Girls**							
**Variable**	**Level**	**Self-harm thoughts at time 1**	**No self-harm thoughts at time 2**	Adjusted OR* (95% CI)	**Overall p-value**	Adjusted OR* (95% CI) from MI	**Multiple adjusted OR**^T^**(95% CI)**
**Alcohol**	**Never/1 or 2x**	316	182	1.0	0.001	1.0	1.0
	**2/4 + per mth**	146	109	2.16 (1.37. 3.39)		2.07 (1.31, 3.27)	1.64 (0.99, 2.71)
**Cannabis**	**Never used**	394	234	1.0	<0.001	1.0	1.0
	**Used**	68	57	3.48 (1.76, 6.90)		2.76 (1.51, 5.06)	2.75 (1.21, 6.21)
**Drugs**	**Never used**	441	275	1.0	0.229	1.0	1.0
	**Used**	21	16	1.88 (0.67, 5.23)		2.07 (0.77, 5.53)	0.50 (0.14, 1.74)
**Bullied**	**Never/1 or 2x**	395	239	1.0	0.006	1.0	1.0
	**2/3 + per mth**	69	53	2.32 (1.27, 4.24)		2.39 (1.31, 4.34)	1.86 (0.96, 3.62)
**Bullying others**	**Never**	360	222	1.0	0.299	1.0	1.0
	**Regularly**	105	70	1.28 (0.81, 2.02)		1.48 (0.95, 2.31)	1.12(0.68, 1.86)
**School connectedness**	**≤24**	201	155	1.0	<0.001	1.0	1.0
	**25+**	266	138	0.32 (0.21, 0.48)		0.30 (0.20, 0.44)	0.36 (0.23, 0.56)
**Alcohol**	**Never/1 or 2x**	132	77	1.0	0.276	1.0	1.0
	**2/4 + per mth**	98	64	1.36 (0.78, 2.37)		1.38 (0.82, 2.32)	1.00 (0.53, 1.90)
**Cannabis**	**Never used**	179	102	1.0	0.023	1.0	1.0
	**Used**	53	40	2.26 (1.12, 4.59)		2.18 (1.17, 4.05)	2.12 (0.88, 5.11)
**Drugs**	**Never used**	216	131	1.0	0.483	1.0	1.0
	**Used**	17	12	1.47 (0.50, 4.36)		1.96 (0.73, 5.25)	0.52 (0.13, 2.03)
**Bullied**	**Never/1 or 2x**	196	116	1.0	0.043	1.0	1.0
	**2/3 + per mth**	37	28	2.34 (1.03, 5.35)		2.51 (1.15, 5.44)	1.53 (0.63, 2.87)
**Bullying others**	**Never**	175	102	1.0	0.084	1.0	1.0
	**Regularly**	59	42	1.76 (0.93, 3.36)		1.35 (0.73, 2.49)	1.84 (0.92, 3.75)
**School connectedness**	**≤24**	115	88	1.0	<0.001	1.0	1.0
	**25+**	120	57	0.27 (0.15, 0.48)		0.26 (0.15, 0.48)	0.30 (0.16, 0.55)

*ORs adjusted for age and trial arm.

^T^ORs adjusted for age, trial arm and all other variables.

MI = Multiple Imputation.

Boys who were regularly bullied were twice as likely to report thoughts of self-harm continuing. For girls, cannabis use increased the odds of self-harming thoughts continuing. Finally, a higher sense of school membership was associated with lower odds of self-harming thoughts and behaviours persisting for girls,

### Help-seeking

Of those who self-harmed, 11% had contacted their GP for problems of anxiety or depression, with help seeking rising to 18% when other professionals were included.

### Associations between self-harming thoughts and behaviour

Of the 5030 participants in the trial, 3942 (78.4%) completed questions on both self-harming thoughts and behaviours at both assessments. Of these, 3125 (79.3%) reported having neither self-harming thoughts nor behaviours at assessment 1. For these young adolescents who did not initially report self-harm, 6.1% reported thoughts only, 1.4% reported behaviours only, whilst 2.6% report both thoughts and behaviours by assessment 2.

Of the 3942 individuals with complete self-harm data, 444 (11.3%) reported self-harming thoughts but no behaviour at assessment 1. Of these, 32.9% reported persistent thoughts (but no behaviour) at assessment 2, while 17.3% also reported the development of self-harming behaviours.

Participants who reported self-harming thoughts at baseline were more likely to develop self-harm behaviour at follow-up than those reporting neither thoughts nor behaviours at baseline (OR = 5.9, 95% CI 4.37-7.86).

## Discussion

Self–harming thoughts and behaviour amongst young adolescents are evident by school year 8 (aged 12–13), with girls in school year 9 (aged 13–14) being at particular risk. Self-harm is common, with one in five reporting thoughts and one in ten at least one act of self-harm over a six month period. These thoughts and behaviours can persist over time, with 60% of those reporting self-harming thoughts and 55% of those reporting self-harming acts doing so across both assessment periods.

Previous studies have focused upon older adolescents and few have included adolescents aged 14 years or younger. A particular strength of our study is the number of young adolescents (≤14 years) we were able to assess (n = 2547). Our results indicate that thoughts and acts of self-harm are evident in those aged ≤14 years. Girls in school year 9 (aged 13/14) were at significantly increased odds of developing thoughts and acts of self-harm than those in year 8 (aged 12/13). This suggests the need for self-harm prevention programmes to be more focused upon younger adolescents (aged 12/13) before self-harming has become established.

Our results with this younger cohort are consistent with the findings from research with older adolescents. Within this older group low mood has been found to increase the likelihood that both boys and girls would develop self-harming thoughts and behaviours and that girls are at increased odds of self-harm than boys [4,21,22]. Similarly our finding of an association between the young adolescents’ relationships and the development and continuity of self-harm over the 12 month period has been noted with older adolescents [10]. In our study insecure peer attachments (i.e. anxious or avoidant) increased the odds of boys and girls developing self-harming behaviours. Similarly being bullied doubled the odds of boys developing self-harming behaviours and for self-harming thoughts persisting. A strong relationship with school was protective with boys being less likely to develop self-harming thoughts and girls being less likely to report continuing self-harming thoughts and behaviours.

In terms of substance use, cannabis use increased the odds of boys developing self-harming thoughts and in these persisting in girls. However, although numbers were small, the greatest risk of developing self-harming behaviours for boys was from the use of illicit street drugs. Boys who used street drugs were six times more likely to develop self-harming behaviour.

Participants were not specifically asked whether they had sought help for self-harm, although help-seeking for emotional problems in general was uncommon. Those who had sought help for anxiety or depression had significantly more symptoms of low mood at baseline and were potentially a more seriously affected group.

Self-harm behaviour at follow up was more likely to be preceded, and then accompanied, by self-harm thoughts. This suggests that focusing upon improving general mental health and developing positive cognitive skills is likely to have an impact on numbers engaging in self-harm behaviours.

### Comparison with other studies

One study to date has investigated self-harm in a community sample of older adolescents (16–17 year olds) using the self-harm questions from the ALSPAC study that we used [31]. In this older age group, 18.8% reported that they had ever self-harmed with rates being significantly higher in females. These findings are consistent with the 15% found in our study with younger adolescents where we also found significantly increased rates of self-harm in girls.

In terms of age, there have been only two previous large prospective studies that have investigated self-harm in community samples of younger (aged 12–15) adolescents, both conducted in Scandanavia [21,22]. Rates varied with one study reporting a lifetime estimate of self-harm of 3% [21], and the other finding 38% of boys and 45% of girls had self-harmed in the past 6 months [22]. These differences may be due to the way self-harm was assessed since single item measures tend to produce lower rates than multiple item questionnaires [32]. We used a single item to assess self-harm. Our 12 month rate of 15% for acts of self-harm reported is significantly higher than the 3% rate previously reported with a similar age group using a single item measure [21]. The definition of self-harm used in both these studies was similar, specifically mentioning an “overdose” or another method of harm. In our study, we also specified “cutting” and this broader definition may have contributed to our higher rates. Alternatively these results may reflect cultural differences or variations in cohort sampling or participant characteristics. Nonetheless our findings suggest that rates of reported self-harm in young adolescents are similar to those of older adolescents.

### Strengths and Limitations

Key strengths include the multi-site design, collection of prospective longitudinal data and high rates of follow-up (78%). The sample is large (n = 3964) and a particular strength is the inclusion of younger adolescents (≤14 years). In terms of limitations, these data were collected from the PROMISE randomised controlled trial, with the primary aim of assessing the effects of school based depression prevention programmes on symptoms of low mood [25]. Although there was no evidence that the trial intervention had any effect on either depression or self-harm behaviour at follow-up [33], we adjusted for trial arm in our analyses presented here. The trial failed to find any significant benefits of a specific cognitive behaviour therapy depression prevention programme over usual school provision. Our study was not therefore specifically designed as a prospective longitudinal survey and as such the assessment of possible self-harm predictors were limited. We were not able to investigate the role of factors such as sexual orientation or smoking that have been shown to be associated with self-harm in adolescents. Secondly we used a sub-set of the self-harm questions from the ALSPAC study. As such, we were unable to determine how many of these thoughts or acts had suicidal intent, the method of self-harm, or provide any insight into the reasons that triggered these thoughts or behaviours. The distinction between acts of self-harm with and without suicidal intent is important but in order to minimise assessment demands in this study our assessment of self-harm was limited. Thirdly, there was no qualitative component to explore potentially important associations. Finally, imputing missing data suggested the potential presence of an association in some instances (e.g. girls being bullied and developing self-harm thoughts), as well as the disappearance of an association in other instances (e.g. girls living arrangement and developing self-harm thoughts). This, along with low prevalence of some of the risk factors (such as the use of illicit drugs) results in imprecise estimates of association, suggests the need for larger, more targeted studies.

Despite these limitations, this study is one of the few to prospectively document the extent of self-harming thoughts and behaviour amongst a large community sample of young adolescents. These findings highlight the importance of developing supportive relationships and the potential need for the widespread provision of preventive initiatives within schools.

## Conclusions

Our findings indicate that self-harm in young adolescents is common and can be persistent, yet few specifically seek help for psychological problems. This suggests a need to raise awareness of self-harm and potential risk factors such as low mood, bullying and drug misuse amongst those who have regular contact with young adolescents in the community, schools and primary care. Training for community staff to understand the prevalence, nature, and factors associated with self-harm might improve better identification. Similarly, training those who have contact with young adolescents in how to talk with them in a calm, open and non-judgemental manner might help to identify those with more persistent thoughts or engaged in more regular self-harming. The development of clear pathways involving child and adolescent mental health services would ensure timely access to specialist assessment and effective treatment for those who require more specialist interventions.

Treatment studies of self-harm in adolescents have traditionally focused upon enhancing cognitive and emotional skills, but the results of recent studies suggest the need to also consider the wider social context [34,35]. Our findings support adopting this broader approach, and suggest that self-harm prevention programmes should promote skills to minimise risk taking behaviour such as cannabis and drug misuse and to facilitate the development of supportive relationships.

Schools offer an accessible and convenient location for the delivery of self-harm prevention programmes which could potentially be widely provided, i.e. universally provided to all children as part of the school curriculum from the age of 12 years. Our results suggest that schools should proactively focus upon reducing bullying and encouraging a positive sense of school membership and belonging, particularly for those young adolescents who feel marginalised. Mental health awareness needs to be raised so that issues such as low mood and self-harm can be openly discussed, local psychological services can be signposted, and access facilitated. Finally, specific advice on and skills to manage the potential risks of drug and cannabis use should be taught.

## Competing interests

No authors have any competing interests that may be relevant to the submitted work.

## Authors’ contributions

We confirm that all authors fulfil the criteria for authorship. PS was principal investigator and will act as guarantor for the paper. RP & KS supervised data collection and MS & AAM undertook the statistical analysis. All authors participated in interpretation of the findings, contributed core ideas and were involved in critically revising the paper for important intellectual content. All authors read and approved the final manuscript.

## Pre-publication history

The pre-publication history for this paper can be accessed here:

http://www.biomedcentral.com/1471-244X/13/328/prepub

## Supplementary Material

Additional file 1 **Table A.** Comparison of socio-demographic, risk factor variables and psychological scores at baseline for those with/without missing information on self-harming (SH) thoughts at baseline and 6 month follow-up. Figures are number (percentage) of participants unless otherwise stated. **Table B**: Comparison of socio-demographic, risk factor variables and psychological scores at baseline for those with/without missing information on self-harming (SH) behaviour at baseline and/or 6 month follow-up. Figures are number (percentage) of participants unless otherwise stated. Click here for file
