# Supplementary material for: Self-harm in young adolescents (12–16 years): onset and short-term continuation in a community sample
Source: BMC Psychiatry. 2013 Dec 2;13:328. doi: 10.1186/1471-244X-13-328 (PMC4219511; doi:10.1186/1471-244X-13-328)
Supplement: Additional file 1 — Table A. Comparison of socio-demographic, risk factor variables and psychological scores at baseline for those with/without missing information on self-harming (SH) thoughts at baseline and 6 month follow-up. Figures are number (percentage) of participants unless otherwise stated. Table B: Comparison of socio-demographic, risk factor variables and psychological scores at baseline for those with/without missing information on self-harming (SH) behaviour at baseline and/or 6 month follow-up. Figures are number (percentage) of participants unless otherwise stated. [file 1471-244X-13-328-S1.docx]

| **Additional file- Table A: Comparison of socio-demographic, risk factor variables and psychological scores at baseline for those with/without missing information on self-harming (SH) thoughts at baseline and 6 month follow-up. Figures are number (percentage) of participants unless otherwise stated.** | | | |  |
| --- | --- | --- | --- | --- |
| **Variable** | **Level** | **Missing SH Thoughts**  N=1066 (21.2 %) | **Not Missing SH Thoughts**  N=3964 (78.8%) | **p-value *** |
| **Gender** | **Male** | 573 (53.8) | 1990 (50.2) |  |
|  | **Female** | 493 (46.2) | 1974 (49.8) | 0.040 |
| **Year Group** | **8** | 244 (22.9) | 1169 (29.5) |  |
|  | **9** | 253 (23.7) | 1141 (28.8) |  |
|  | **10** | 288 (27.0) | 1036 (26.1) |  |
|  | **11** | 281 (26.4) | 618 (15.6) | <0.001 |
| **Ethnicity** | **White** | 566 (86.3) | 3352 (85.4) |  |
|  | **Other** | 90 (13.7) | 575 (14.6) | 0.535 |
| **Living Situation** | **Both parents** | 364 (55.7) | 2608 (67.0) |  |
|  | **Parent & Partner** | 93 (14.2) | 513 (13.2) |  |
|  | **Single Parent** | 176 (26.9) | 712 (18.3) |  |
|  | **Other** | 21 (3.2) | 61 (1.6) | <0.001 |
| **Alcohol** | **Never/1 or 2x** | 397 (70.8) | 3229 (82.1) |  |
|  | **2/4 + per month** | 164 (29.2) | 705 (17.9) | <0.001 |
| **Cannabis** | **Never used** | 464 (82.0) | 3633 (92.3) |  |
|  | **Used** | 102 (18.0) | 302 (7.7) | <0.001 |
| **Street Drugs** | **Never used** | 546 (97.7) | 3860 (98.0) |  |
|  | **Used** | 13 (2.3) | 78 (2.0) | 0.588 |
| **Bullying Others** | **Never** | 461 (78.8) | 3269 (83.2) |  |
|  | **Ever** | 124 (21.2) | 661 (16.8) | 0.009 |
| **Bullied** | **Never/1 or 2x** | 507 (90.5) | 3640 (92.3) |  |
|  | **2/4 + per month** | 53 (9.5) | 303 (7.7) | 0.144 |
| **Self-harm Behaviour** | **No** | 499 (89.4) | 3581 (90.5) |  |
|  | **Yes** | 59 (10.6) | 376 (9.5) | 0.422 |
| **Own Car** | **No** | 92 (12.2) | 233 (5.9) |  |
|  | **Yes** | 664 (87.8) | 3689 (94.1) | <0.001 |
| **Own Room** | **No** | 126 (16.7) | 594 (15.2) |  |
|  | **Yes** | 628 (83.3) | 3318 (84.8) | 0.288 |
| **Holiday** | **No** | 134 (17.7) | 575 (14.7) |  |
|  | **Yes** | 621 (82.3) | 3337 (85.3) | 0.089 |
| **Own a PC** | **No** | 16 (2.1) | 44 (1.1) |  |
|  | **Yes** | 738 (97.9) | 3870 (98.9) | 0.079 |
| **Baseline MFQ** | **0-4** | 526 (63.5) | 2826 (71.4) |  |
|  | **5+** | 302 (36.5) | 1130 (28.6) | <0.001 |
| **Attachment** | **Secure** | 462 (85.2) | 3079 (84.1) |  |
|  | **Avoidant** | 48 (8.9) | 335 (9.2) |  |
|  | **Anxious** | 32 (5.9) | 247 (6.7) | 0.733 |
| **GP for anx/depression** | **No** | 361 (93.8) | 3629 (96.2) |  |
|  | **Yes** | 24 (6.2) | 143 (3.8) | 0.020 |
| **Seen anyone for anx/depression** | **No** | 532 (91.1) | 3667 (93.5) |  |
|  | **Yes** | 52 (8.9) | 254 (6.5) | 0.030 |
|  |  |  |  |  |
| **Low School Connectedness** | **No** | 480 (76.6) | 3253 (82.6) |  |
|  | **yes** | 147 (23.4) | 687 (17.4) | <0.001 |
| **SMFQ, mean (SD)** | | 4.5 (5.2) | 3.7 (4.8) | <0.001 |
|  | |  |  |  |
| **School Connectedness, mean (SD)** |  | 29.5 (6.6) | 30.6 (6.2) | <0.001 |
|  |  |  |  |  |
|  |  |  |  |  |
|  |  |  |  |  |
|  |  |  |  |  |
|  |  |  |  |  |
|  | |  |  |  |
|  |  |  |  |  |

*** Chi-squared test for r x 2 tables**

| **Additional file - Table B: Comparison of socio-demographic, risk factor variables and psychological scores at baseline for those with/without missing information on self-harming (SH) behaviour at baseline and/or 6 month follow-up. Figures are number (percentage) of participants unless otherwise stated.** | | | |  |
| --- | --- | --- | --- | --- |
| **Variable** | **Level** | **Missing SH Behaviour**  N=1075 (21.4 %) | **Not Missing SH Behaviour**  N=3955 (78.6%) | **p-value *** |
| **Gender** | **Male** | 577 (53.7) | 1986 (50.2) |  |
|  | **Female** | 498 (46.3) | 1969 (49.8) | 0.044 |
| **Year Group** | **8** | 244 (22.7) | 1169 (29.6) |  |
|  | **9** | 261 (24.3) | 1133 (28.6) |  |
|  | **10** | 286 (26.6) | 1038 (26.2) |  |
|  | **11** | 284 (26.4) | 615 (15.5) | <0.001 |
| **Ethnicity** | **White** | 574 (86.4) | 3344 (85.3) |  |
|  | **Other** | 90 (13.6) | 575 (14.7) | 0.449 |
| **Living Situation** | **Both parents** | 366 (55.2) | 2606 (67.1) |  |
|  | **Parent & Partner** | 95 (14.3) | 511 (13.2) |  |
|  | **Single Parent** | 180 (27.1) | 708 (18.2) |  |
|  | **Other** | 22 (3.3) | 60 (1.5) | <0.001 |
| **Alcohol** | **Never/1 or 2x** | 404 (71.0) | 3222 (82.1) |  |
|  | **2/4 + per month** | 165 (29.0) | 704 (17.9) | <0.001 |
| **Cannabis** | **Never used** | 468 (81.7) | 3629 (92.4) |  |
|  | **Used** | 105 (18.3) | 299 (7.6) | <0.001 |
| **Street Drugs** | **Never used** | 553 (97.5) | 3853 (98.0) |  |
|  | **Used** | 14 (2.5) | 77 (2.0) | 0.420 |
| **Bullying Others** | **Never** | 467 (78.5) | 3263 (83.2) |  |
|  | **Ever** | 128 (21.5) | 657 (16.8) | 0.004 |
| **Bullied** | **Never/1 or 2x** | 514 (90.3) | 3633 (92.3) |  |
|  | **2/4 + per month** | 55 (9.7) | 301 (7.7) | 0.096 |
| **Self-harm Thoughts** | **No** | 455 (79.5) | 3181 (80.7) |  |
|  | **Yes** | 117 (20.5) | 763 (19.3) | 0.532 |
| **Own Car** | **No** | 94 (12.3) | 231 (5.9) |  |
|  | **Yes** | 671 (87.7) | 3682 (94.1) | <0.001 |
| **Own Room** | **No** | 125 (16.4) | 595 (15.2) |  |
|  | **Yes** | 638 (83.6) | 3308 (84.8) | 0.426 |
| **Holiday** | **No** | 139 (18.2) | 570 (14.6) |  |
|  | **Yes** | 625 (81.8) | 3333 (85.4) | 0.039 |
| **Own a PC** | **No** | 18 (2.4) | 42 (1.1) |  |
|  | **Yes** | 745 (97.6) | 3863 (98.9) | 0.004 |
| **Baseline MFQ** | **0-4** | 530 (63.2) | 2822 (71.5) |  |
|  | **5+** | 308 (36.8) | 1124 (28.5) | <0.001 |
| **Attachment** | **Secure** | 472 (85.4) | 3069 (84.1) |  |
|  | **Avoidant** | 48 (8.7) | 335 (9.2) |  |
|  | **Anxious** | 33 (6.0) | 246 (6.7) | 0.721 |
| **GP for anx/depression** | **No** | 370 (94.1) | 3620 (96.2) |  |
|  | **Yes** | 23 (5.9) | 144 (3.8) | 0.052 |
| **Seen anyone for anx/depression** | **No** | 537 (90.6) | 3662 (93.6) |  |
|  | **Yes** | 56 (9.4) | 250 (6.4) | 0.006 |
| **Low School Connectedness** | **No** | 486 (76.4) | 3247 (82.6) |  |
|  | **yes** | 150 (23.6) | 684 (17.4) | <0.001 |
|  |  |  |  |  |
|  | |  |  |  |
| **SMFQ, mean (SD)** |  | 4.5 (5.2) | 3.7 (4.7) | <0.001 |
|  |  |  |  |  |
| **School Connectedness, mean (SD)** |  | 29.5 (6.7) | 30.6 (6.2) | <0.001 |
|  |  |  |  |  |
|  |  |  |  |  |
|  |  |  |  |  |
|  |  |  |  |  |
|  | |  |  |  |
|  |  |  |  |  |

*** Chi-squared test for r x 2 tables**
